# ﻿A new species of medicinal leech in the genus *Hirudo* Linnaeus, 1758 (Hirudiniformes, Hirudinidae) from Tianjin City, China

**DOI:** 10.3897/zookeys.1095.74071

**Published:** 2022-04-13

**Authors:** Hao Wang, Fan-Ming Meng, Si-Jie Jin, Jiang-Wei Gao, Xiang-Rong Tong, Zi-Chao Liu

**Affiliations:** 1 Engineering Research Center for Exploitation & Utilization of Leech Resources in Universities of Yunnan Province, School of Agriculture & Life Sciences, Kunming University, Kunming 650214, China Universities of Yunnan Province Kunming China; 2 Department of Parasitology, Xiangya School of Medicine, Central South University, Changsha 430001, China Central South University Changsha China

**Keywords:** Blood-feeding leech, COI, molecular phylogeny, new species, taxonomy

## Abstract

Medicinal leeches in the genus *Hirudo* have been utilized for therapeutic procedures for thousands of years. To date, six known species of *Hirudo* are widely distributed in different regions of the Eurasian continent. In this study, a new medicinal leech species *Hirudotianjinensis* Liu, **sp. nov.** is described based upon specimens collected from Tianjin City, China. The new species can be distinguished from its congeners by a combination of characters: blackish green dorsum with five continuous yellow longitudinal stripes; six sensillae on dorsal annulus a2 of segments VIII–XXV; greyish green ventrum with irregular bilateral dark brown spots; dorsum and abdomen separated by a pair of pale yellow stripes; front half atrium wrapped by white prostate; apparent albumen gland; epididymis massive in relation to ejaculatory bulb. The phylogenetic tree based upon COI implies a sister relationship to *H.nipponia* Whitman, 1886. A key to the known species is provided.

## ﻿Introduction

Leeches are carnivorous, hermaphroditic, and wormlike invertebrates with a sucker at each end of their bodies, belonging to the class Hirudinea of phylum Annelida. Approximately 650–680 leech species belonging to four subclasses, five orders, 13 families, and 149 genera have been nominated worldwide (Sawyer 1986; Sket and Trontelj 2008), of which 116 species are distributed in China. Natural habitats are predominantly fresh water and occasionally grassland, soil, or ocean water.

“Medicinal leech” is a common name referring to a group of aquatic blood-feeding ectoparasitic leech species traditionally employed for treating such a large variety of human diseases as amenorrhea, osteoarthritis, trauma, and blood stasis syndrome (Sig et al. 2017; Wang, et al. 2018). In the modern era, it also serves as important model systems for understanding the structure, function, development, regeneration, and repair of nervous systems (Herlin et al. 2017; Liu, et al. 2018). Most medicinal leech species belong to genus *Hirudo* of family Hirudinidae of which the most widely used are *H.medicinalis* Linnaeus, 1758 and *H.nipponia* Whitman, 1886 with wide distributions in the European Palaearctic and Sino-Japanese regions, respectively. In addition, a few species belonging to genus *Poecilobdella* of family Hirudinidae e.g., *P.manillensis* (Lesson, 1842) and genus *Whitmania* of family Haemopidae e.g., *W.pigra* (Whitman, 1884) are also used as medicine in some East and Southeast Asian countries. The salient characteristics of *Hirudo* are as follows: the vagina bears a small caecum; there is no vaginal duct present; there are no/few salivary papillae; and there is no furrow on upper lip (Sawyer 1986). To date, six species of this genus have been described: *H.nipponia*, *H.medicinalis*, *H.verbena* Cerena, 1820, *H.orientalis* Utevsky, 2005, *H.troctina* Johnson, 1816, and *H.sulukii* Shain, 2016 (Utevsky and Trontelj 2005; Saglam et al. 2016). Among these, *H.nipponia* is distributed in eastern Russia, China, Japan, and Mongolia while the other five species are endemic in Europe, North Africa, and western Asia.

As a well-known medicinal leech species, *H.nipponia* has been reported in China for many decades (Gee and Wu 1926). It is widely distributed in most areas of China except for Xinjiang and Tibet Autonomous Regions (Yang 1996). Reputed as an important traditional Chinese medicine, it has been recorded in Chinese Pharmacopoeia for the treatments of blood stasis, amenorrhea, edema, apoplexy, hemiplegia, and trauma (Committee 2020). In ancient folklore, medicinal leech therapy had been also widely applied for varicosity and arthrolithiasis by sucking blood from diseased sites using this leech species.

Located in North China, Tianjin City is one of the four major municipalities directly under the central Chinese government. It is one of the major areas producing medicinal leeches such as *H.nipponia* and *W.pigra* (Whitman, 1884) in China. In a recent collection, a total of 30 medicinal leech specimens differed significantly in morphology from the other *Hirudo* species collected from Tianjin City. These specimens represent a new species of *Hirudo*.

## ﻿Materials and methods

### ﻿Specimen sampling and morphological observation

**Figure 1.**
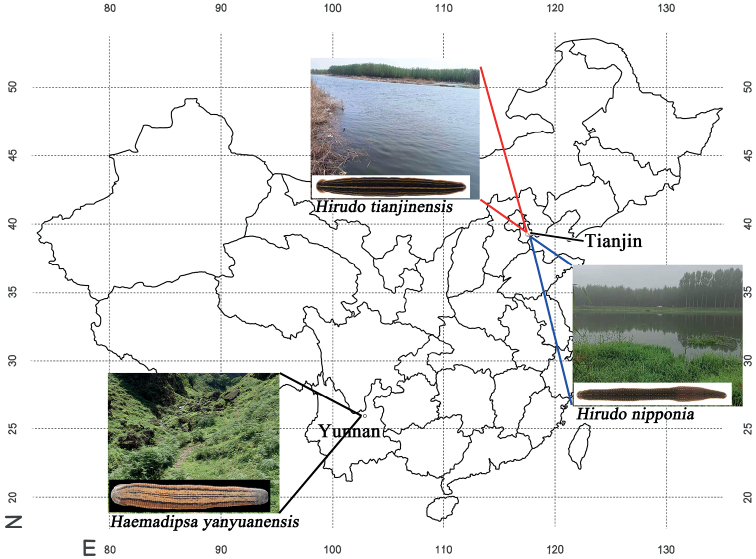
Map of geographic collecting locations for three species of Chinese leeches. *H.tianjinensis* and *H.nipponia* were collected in adjacent areas of Tianjin City, and *Haemadipsayanyuanensis* was collected in Yunnan Province.

On 19 August 2020, a total of 30 specimens described herein was captured in Caobai River, Haogezhuang Town, Baodi District, Tianjin City, China (39°36'40"N, 117°23'13"E). After relaxing in 15% ethanol, leeches were fixed with 95% ethanol for preservation, measurement, dissection, and molecular analysis. All type specimens (one holotype and 16 paratypes) were measured with a digital caliper with an accuracy of 0.1 mm. Six specimens were used for anatomical observation. The female and male reproductive systems were observed after dissecting along dorsal midline and fixing with insect needles on a wax tray. Jaws and teeth inside the anterior sucker were observed after cutting along the ventral midline of the anterior sucker. Morphological traits of the holotype specimen were observed and photographed by a stereomicroscopy with digital camera. To determine the taxonomy, seven specimens of the new species, two specimens of its sister species *H.nipponia* from Tianjin City (39°28'04"N, 117°28'48"E), and one specimen of the outgroup species *Haemadipsayanyuanensis* Liu et Song, 1977 from Yunnan Province (26°02'35"N, 102°49'34"E) were utilized for molecular studies (Fig. 1). Voucher specimens were deposited at the Engineering Research Center for Exploitation & Utilization of Leech Resources in Universities of Yunnan Province, School of Agriculture & Life Sciences, Kunming University, Kunming.

### ﻿DNA extraction, PCR, and DNA sequencing

Caudal suckers were removed with a scalpel and immediately ground to powder in liquid nitrogen. The objective of selecting caudal sucker tissue was to avoid contamination from the gut contents of DNA from unknown host blood and various microorganisms. According to the manufacturer’s instructions, genomic DNA was extracted with Universal DNA kit (Mei5bio, China).

Mitochondrial cytochrome c oxidase subunit I (COI) fragments were amplified by polymerase chain reaction (PCR) with primers LCO 1490 and HCO 2198 (Folmer et al. 1994). PCR program was as follows: 2 min at 94 °C followed by 30 cycles of 30 s at 92 °C, 45s at 55 °C, 60 s at 72 °C, and a final extension step of 10 min at 72 °C. All PCR products were purified by elution from 1% agarose gel and then submitted to Qingke Biotech for bi-directional sequencing on an Applied Biosystems DNA sequencer (ABI 3730XL, USA).

### ﻿Phylogenetic analyses

Prior to further analysis, DNA sequences generated here were deposited into the database of GenBank with the following accession numbers: MZ820656–MZ820659 for *H.tianjinensis*; MZ820661 and MZ820662 for its sister species *H.nipponia*; MZ820660 for *Haemadipsayanyuanensis* as outgroup. In addition, three sequences of *Whitmania* were also included here for its close relationship with *Hirudo*, which were reported in previous studies (Phillips and Siddall 2009; Nikitina et al. 2016). A total of 29 *Hirudo*COI sequences from GenBank were also downloaded for phylogenetic analysis (Table 1).

**Table 1.** Locality with geographic coordinates and GenBank accession numbers of specimens for phylogenetic analysis.

Species	Voucher ID	Locality	Coordinates	GenBank Acc. No.	References
*H.tianjinensis*	20200251 20200254 20200256	Tianjin, CN	39°36'40"N, 117°23'13"E	MZ820656	This study
	20200252 20200253	Tianjin, CN	39°36'40"N, 117°23'13"E	MZ820657	This study
	20200255	Tianjin, CN	39°36'40"N, 117°23'13"E	MZ820658	This study
	20200257	Tianjin, CN	39°36'40"N, 117°23'13"E	MZ820659	This study
*H.nipponia*	20200301	Tianjin, CN	39°28'04"N, 117°28'48"E	MZ820661	This study
	20200302	Tianjin, CN	39°28'04"N, 117°28'48"E	MZ820662	This study
		KR		AY763153	(Trontelj and Utevsky 2005)
		KR		AY425450	(Borda and Siddall 2004)
		KR		GQ368749	(Phillips and Siddall 2009)
*H.medicinalis*		Halle, DE		AY763148	(Trontelj and Utevsky 2005)
	SMNH111543	Gasavartar, SE	57°40'52"N, 18°35'35"E	HQ333519	(Kvist et al. 2010)
	HR20	Hrast, SI		EF446712	(Siddall et al. 2007)
		FR		EU100093	(Borda et al. 2008)
		Saratov, RU	51°91'03"N, 47°34'90"E	KU672396	(Nikitina et al. 2016)
*H.orientalis*	COIH03	Urmia, IR	37°32'51.09"N, 56°22'56.72"E	KY989464	(Darabi-Darestani et al. 2018)
		Agdam, AZ		AY763154	(Trontelj and Utevsky 2005)
		Samarkand, UZ		EF405599	(Utevsky et al. 2007)
		AZ		GQ368750	(Phillips and Siddall 2009)
*H.verbana*		Stavropol, RU	49°01'09"N, 43°48'21"E	KU672397	(Nikitina et al. 2016)
	HV3	Bursa, TR	40°10'23"N, 28°37'26"E	KU216244	(Saglam et al. 2016)
	HV19	Samsun, TR	41°34'48"N, 36°04'31"E	KT692947	(Saglam et al. 2016)
	H2P	Galicia, ES		MT797290	(Arias et al. 2021)
	17(1)	Kherson, UA		JN104644	(Trontelj and Utevsky 2012)
	13(1)	Izmir, TR		JN083804	(Trontelj and Utevsky 2012)
		Giessen, DE		EF125043	(Kutschera et al. 2007)
		Ohrid, MK		AY763150	(Trontelj and Utevsky 2005)
	KA46	Povir, SI		EF446701	(Siddall et al. 2007)
*H.troctina*		Marrakech, MA		AY763155	(Trontelj and Utevsky 2005)
	HT56	Lebna Dam, TN		JQ364946	(Trontelj and Utevsky 2012)
	H28b	AZ		GQ368751	(Phillips and Siddall 2009)
*H.sulukii*	HS1	Adiyaman, TR	37°59'35"N, 38°48'52"E	KU216239	(Saglam et al. 2016)
	HS2	Adiyaman, TR	37°59'35"N, 38°48'52"E	KU216240	(Saglam et al. 2016)
	HS5	Gaziantep, TR	37°18'12"N, 37°14'53"E	KU216241	(Saglam et al. 2016)
	HS6	Batman, TR	37°51'46"N, 41°01'00"E	KU216242	(Saglam et al. 2016)
	HS7	Batman, TR	37°51'46"N, 41°01'00"E	KU216243	(Saglam et al. 2016)
*W.acranulata*				NC023928	
*W.pigra*				MN729556	
*W.laevis*		Shaanxi, CN	32°43"N, 108°46"E	KM655839	(Ye et al. 2015)
*Haemadipsayanyuanensis*	20200351	Yunnan, CN	26°02'35"N, 102°49'34"E	MZ820660	This study

*ISO country codes: AZ, Azerbaijan; CN, China; DE, Germany; ES, Spain; FR, France; IR, Iran; KR, Korea; MA, Morocco; MK, Former Yugoslav Republic of Macedonia; RU, Russia; SE, Sweden; SI, Slovenia; TN, Tunisia; TR, Turkey; UA, Ukraine, and UZ, Uzbekistan.

Sequences were aligned and edited using ClustalW implemented in MEGA7 (Kumar et al. 2016). The dataset of COI gene was used for phylogenetic tree construction using Maximum-Likelihood (ML) and Bayesian-Inference (BI) approaches with *Haemadipsayanyuanensis* as the outgroup. ML analysis was conducted using 1000 ML bootstrap replications. BI analysis was performed in program MrBayes 3.2.6 with Markov chain Monte Carlo analysis (MCMC) in two parallel runs and with four chains each (Ronquist et al. 2012). Chain length was set as 1,500,000 generations and sampled every 1000 generations during calculations. Genetic divergences based upon the COI sequences were calculated for depicting evolutionary divergence between *Hirudo* species using uncorrected p-distances as implemented in MEGA 7 (Kumar et al. 2016).

## ﻿Results

### ﻿Taxonomy

**Family Hirudinidae Whitman, 1886**

#### Genus *Hirudo* Linnaeus, 1758

##### ﻿ Hirudo tianjinensis

Taxon classificationAnimaliaArhynchobdellidaHirudinidae

Liu sp. nov.

40F2FCEA-4388-55A8-A0BE-517976434483

http://zoobank.org/Fdf1eb12-A436-4e50-B15e-Dc0324773443

###### Material examined.

**Figure 2.**
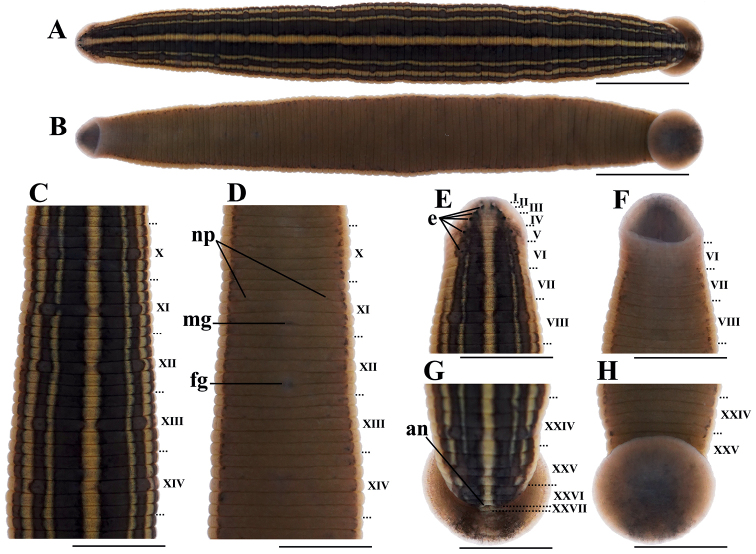
External morphology of *H.tianjinensis* holotype **A** dorsal and **B** ventral view of whole body **C** dorsal and **D** ventral view of somites X–XIV **E** dorsal and **F** ventral view of somites I–VIII **G** dorsal and **H** ventral view of somites XXIV–XXVII and caudal sucker. Abbreviations: an, anus; e, eye; fg, female gomopore; mg, male gomopore; np, nephridiopores. Scale bars: 5 mm (**A, B**), 2.5 mm (**C–H**).

**Figure 3.**
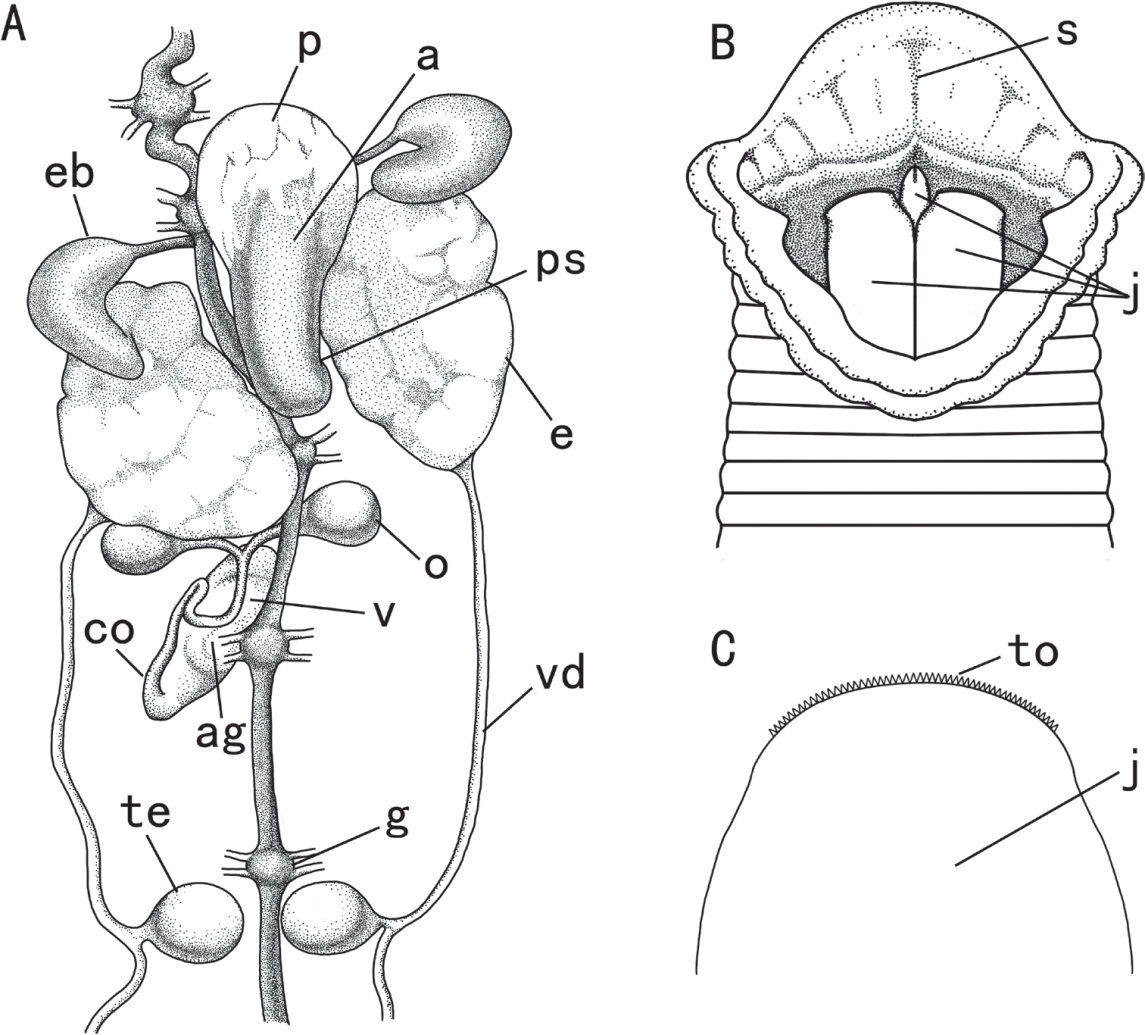
Internal characters of *H.tianjinensis***A** dorsal view of reproductive system **B** ventral view of dissected anterior sucker **C** ventral view of jaw. Abbreviations: a, atrium; ag, albumen gland; co, common oviduct; e, epididymis; eb, ejaculatory bulb; g, ganglion; j, jaw; o, ovisac; p, prostate; ps, penis sheath; s, sulcus; te, testisac; to, tooth; v, vagina; vd, vas deferens.

***Holotype*.** 20200231; Engineering Research Center for Exploitation & Utilization of Leech Resources in Universities of Yunnan Province, School of Agriculture & Life Sciences, Kunming University, Kunming; Body length 31.2 mm, maximal body width 3.6 mm, width of anterior sucker 1.8 mm, width of posterior sucker 3.3 mm; Caobai River, Haogezhuang Town, Baodi District, Tianjin City, China; 39°36'40"N, 117°23'13"E, 5 m; collected by Zichao Liu, 19 Aug. 2020 (Figs 1, 2, 3). ***Paratypes*.** 16 ex.; collected information same as holotype; 20200232–20200247, Engineering Research Center for Exploitation & Utilization of Leech Resources in Universities of Yunnan Province, School of Agriculture & Life Sciences, Kunming University, Kunming.

###### Diagnosis.

*Hirudotianjinensis* can be distinguished from its congeners by the following combination of characters: blackish green dorsum with five continuous yellow longitudinal stripes; six sensillae on dorsal annulus a2 of segments VIII–XXV making dorsal golden midline notched, and two lateral blackish green dorsal line rosary; greyish green ventrum with irregular dark brown spots bilaterally; dorsum and abdomen separated by a pair of pale yellow stripes; front half of atrium wrapped by white prostate; apparent albumen gland; epididymis massive in relation to ejaculatory bulb.

###### Description.

Blood-feeding aquatic leech, medium body size, length 34.8 ± 3.5 mm (n = 17), maximum body width 3.7 ± 0.4 mm, width of anterior sucker 1.8 ± 0.2 mm, width of caudal sucker 3.6 ± 0.4 mm. Caudal sucker diameter slightly narrower than maximal body breadth.

In relaxed state, dorsum and abdomen flat and willow-leaf like. Blackish green dorsum with five continuous yellow stripes. Dorsal midline widest, extending from the first to the last somite. Yellow stripes separate dorsum into six blackish green longitudinal stripes of which the middle two are the widest and the lateral four are narrower. Six sensillae on middle a2 of segments VIII–XXV making middle golden stripe notched and two lateral blackish green stripes rosary. Venter greyish green with irregular dark brown spots bilaterally edged by a pair of pale yellow stripes. No visible sensillae in abdomen. Caudal sucker reddish brown, with dark dorsum and pale-colored abdomen.

Complete somite five-annulate, numbers of annuli per somite: I–III: one, IV–V: two, VI–VII: three, VIII: four, IX–XXIII: five (b1, b2, a2, b5, b6), XXIV: four, XXV: three, XXVI–XXVII: two, in total: 27 somites and 103 annuli.

Wide mouth in white anterior sucker. Jaws trignathous, one in the middle and one on each side, 55–67 horny teeth in each jaw. Five pairs of eyes almost circular or irregular shaped on annulus 2, 3, 4, 6, and 9. Fifth pair of eyes smallest and sometimes difficult to observe. Two nephridiopores in submarginal annulus b2 of each complete somite. Gonopores situated in furrow between annuli, separated by five annuli, male pore in furrow XI b5/b6 (annulus 31/32), female pore in furrow XII b5/b6 (annulus 35/36). Anus in middle dorsum between the last two annuli.

Male reproductive system: pyriform atrium large, located at venter of ganglion in segment XI; prostate white and developed, covering front half of atrium, strikingly different from its sister species *H.nipponia* whose prostate is almost invisible; penis sheath with a duct bent anteriorly in segment XI; epididymis massive in relation to ejaculatory bulb, tightly packed masses of ducting standing upright on either atrial side; ejaculatory bulb tapering sharply and connected to dorsal epididymis; testisacs ovoid, 11 pairs, in segments XIII–XXIII.

Female reproductive system: composed usually of one vagina, two ovisacs, and a curved duct in segments XII–XIII; vagina long, upright, and ellipsoidal with apparent albumen gland on middle surface, no vaginal duct; ovisacs ovoid, smaller than testisacs, connected to vagina via a curved common oviduct.

###### Remarks.

This species is frequently confused with *H.nipponia* due to their morphological similarities and overlapping distributions. Local villagers often mistook it for *H.nipponia* for therapeutic usage. However, they can be distinguished using a series of morphological characteristics such as color pattern, number of sensillae, and reproductive system morphology (Table 2).

**Table 2.** Morphological comparison between *H.tianjinensis* and *H.nipponia*.

Traits	*H.tianjinensis*	*H.nipponia*
Sensillae	six sensillae in dorsum, absent in abdomen	six sensillae both in dorsum and abdomen
Stripes on dorsal surface	five yellow continuous longitudinal stripes	five yellow dotted longitudinal stripes, yellow spots on a2 weak or absent
Prostate	developed, wrapping front-half of atrium	absent
Ejaculatory bulb	tapering sharply	tapering gradually

###### Etymology.

The specific name *tianjinensis* is derived from Tianjin City, a municipality directly under the central government in China, where type specimens were collected.

###### Distribution.

*Hirudotianjinensis* was collected from Caobai River, Haogezhuang Town, Baodi District, Tianjin City, China, which flows into Bohai Sea. Water was moderately polluted. Aquatic plants and irregular pumice stones were abundant along the riverbed. This species normally hid under pumice stones. Whenever people and animals pass by, it quickly adsorbs on their ankles for sucking blood.

### ﻿Molecular phylogeny and genetic divergence

**Figure 4.**
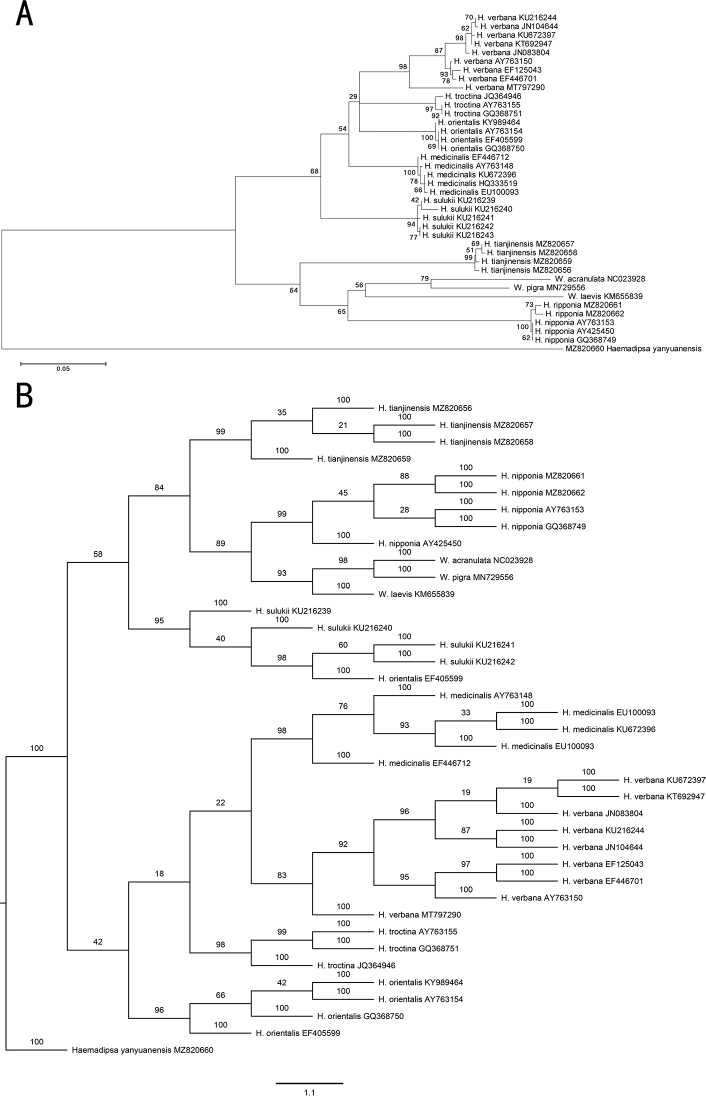
Phylogenetic analysis of *H.tianjinensis* with other species of *Hirudo***A** maximum-likelihood tree was constructed using MEGA 7 with 1000 ML bootstrap replications **B** bayesian inference tree was constructed using MrBayes with four Markov chains. Chain length was set as 1,500,000 generations and sampled every 1000 generations.

Phylogenetic analysis was performed based upon available COI sequences of *Hirudo* from NCBI. Generally, sequences of each species were all well clustered. The results of ML and BI trees were largely similar to each other with the exception of *H.sulukii*. The newly reported species *H.tianjinensis* was clustered with *H.nipponia* and species of *Whitmania* and formed a single branch separated from the other species. This result was consistent with previous phylogenetic analysis on *Hirudo* and revealed the *Hirudo* a paraphyletic group (Fig. 4) (see also Phillips and Siddall 2009; Ye et al. 2015; Nikitina et al. 2016).

The average genetic divergence based upon uncorrected p-distances was also performed. In general, the distance of intra-species was < 1.5% except for sequences of *H.verbena* (range 0.2% to 6.4%). The intraspecific distance of the new species *H.tianjinensis* ranged from 0.2% to 0.9%. And the interspecific distance between *H.tianjinensis* and *H.nipponia* was 16.7% (Table 3).

**Table 3.** Interspecific genetic divergence between seven *Hirudo* species and intraspecific difference within each species based upon analyses of their COI sequences (uncorrected p-distance: %±SD).

Species	Intraspecific range & SD	Interspecific mean ±SD					
*H.tianjinensis*	0.2–0.9±0.2						
*H.nipponia*	0–0.6±0.4	16.7±0.2					
*H.medicinalis*	0–0.6±0.2	18.1±0.2	19.3±0.2				
*H.orientalis*	0–0.2±0.1	16.5±0.1	20.7±0.0	8±0.2			
*H.verbana*	0.2–6.4±2	19.3±0.6	19.3±0.5	9.3±0.5	8.7±0.4		
*H.troctina*	0–0.9±0.5	17.7±0.2	18.5±0.1	8.9±0.1	8.7±0.1	9.5±0.5	
*H.sulukii*	0–1.5±0.6	17.2±0.2	19.6±0.5	9.6±0.4	10.6±0.3	11.3±0.4	11.3±0.3

### ﻿A key to the seven species of *Hirudo*

**Table:** 

1	Dorsum with five longitudinal yellow or greenish yellow stripes	**2**
–	Dorsum with two or four reddish or orange stripes	**3**
2	Dorsum with five continuous yellow or greenish yellow stripes; prostate apparent	***H.tianjinensis* sp. nov.**
–	Dorsum with five yellow dotted stripes; prostate absent	***H.nipponia***
3	Epidimymis massive in relation to ejaculatory bulb	**4**
–	Epidimymis small/medium-sized in relation to ejaculatory bulb	**6**
4	Vagina terminally folded	***H.medicinalis***
–	Vagina long and upright	**5**
5	Dorsum green, two orange paramedian stripes thin; venter black, irregularly arranged and sized black markings	***H.troctina***
–	Dorsum olive green, two orange paramedian stripes thick; venter pale greenish, and covered with small number of irregular black markings	***H.sulukii***
6	Dorsum with broad, pale orange diffuse paramedian stripes; vagina sharply folded; epidimysis small, not much larger than eiaculatory bulb	***H.verbana***
–	Dorsum with thin, deep orange colored paramedian stripes; vagina evenly curved; epidimysis medium-sized, somewhat larger than ejaculatory bulb	***H.orientalis***

## ﻿Discussion

Six species of *Hirudo* have been identified globally. Except *H.nipponia*, the other five species are distributed in Europe and the Middle East, usually referred to as the European leeches. Their diagnostic features and species differences were described in detail (Utevsky and Trontelj 2005; Saglam 2019). The results of molecular taxonomy showed that they are closely correlated. *Hirudonipponia* is easily distinguished from the remaining five species in morphology, because the former is obviously smaller and has five longitudinal stripes in dorsum. *Hirudonipponia* is mainly distributed in East Asia, including Japan, Mongolia, the Russian Far East, and most of China.

*Hirudotianjinensis* has been collected from Tianjin City, a northern Chinese metropolis. It belongs to *Hirudo* based upon a series of morphological characteristics (Sawyer 1986). The detailed morphological comparison between *H.tianjinensis* and *H.nipponia* were obtained by combining specimen observation and previous descriptions (Whitman 1886; Lai and Chen 2010). *Hirudotianjinensis* is similar to *H.nipponia* in morphology but with some distinct differences in color pattern and reproductive system morphology (Table 2). When they were cultured together, *H.tianjinensis* often sucked on the blood of *H.nipponia* while the latter never sucked that of the former. The results of molecular taxonomy showed that *H.tianjinensis* is a sister species to *H.nipponia*. In geographical distribution, *H.tianjinensis* and *H.nipponia* partially overlap. *Hirudonipponia* is found in the whole distribution area of *H.tianjinensis*, indicating that there is probably a common origin between the two species.

Although most medical leeches can bite people, causing continuous bleeding, inflammation, itching, and even bacterial infection, the major reason for attracting people’s attention lies in their important medical values. In China, medicinal application of leeches was first recorded in Shennong’s Herbal Classic 2,000 years ago. In the modern era, Chinese Pharmacopoeia recommends three leech species, *H.nipponia*, *W.pigra*, and *W.acranulata* (Whitman, 1886) for treating arthritis, stroke, myocardial infarction, and amenorrhea (Committee 2020). *Hirudonipponia* is the most widely utilized medicinal leech species with the strongest antithrombin activity compared with the other two medicinal leech species listed in Chinese Pharmacopoeia. Nearly 2,000 tons of leeches are freeze-dried for medicinal uses with an annual value of 1.4 billion US dollars. Due to the importance in medicine, wild-type *H.nipponia* have been overly harvested, and thus become somewhat endangered. For the purposes of sustainable utilization of medical leech resources and species protection, it is imperative to study the medical leech.

The medical leech has important clinical applications since it contains many of bioactive components with the functions of anticoagulant, thrombolytic, matrix degradation analgesic, and anti-inflammatory, such as hirudin, antistasin, and hyaluronidase (Sig et al. 2017; Kwak et al. 2019; Muller et al. 2020). More than 20 molecules and their mechanistic modes have been identified, and more unique active ingredients of leeches await discovery.

Although the medical leech has important medical value, its taxonomic research is rather behind. Traditional practitioners tend to refer to *H.nipponia* and *H.tianjinensis* as the linear and dotted leeches respectively in Tianjin City. In this work, *H.tianjinensis* is formally named as a new species and its medical value needs to be further studied in the future.

## Supplementary Material

XML Treatment for Hirudo tianjinensis
